# Circulating tumor DNA detection in MRD assessment and diagnosis and treatment of non-small cell lung cancer

**DOI:** 10.3389/fonc.2022.1027664

**Published:** 2022-10-27

**Authors:** Xiaoxu Fang, Shaokun Yu, Yingying Jiang, Yan Xiang, Kaihua Lu

**Affiliations:** Department of Oncology, The First Affiliated Hosptial of Nanjing Medicial University, Nanjing, China

**Keywords:** ctDNA, liquid biopsy, NSCLC, therapy monitoring, minimal residual disease (MRD)

## Abstract

Circulating tumor DNA (ctDNA) has contributed immensely to the management of hematologic malignancy and is now considered a valuable detection tool for solid tumors. ctDNA can reflect the real-time tumor burden and be utilized for analyzing specific cancer mutations *via* liquid biopsy which is a non-invasive procedure that can be used with a relatively high frequency. Thus, many clinicians use ctDNA to assess minimal residual disease (MRD) and it serves as a prognostic and predictive biomarker for cancer therapy, especially for non-small cell lung cancer (NSCLC). Advanced methods have been developed to detect ctDNA, and recent clinical trials have shown the rationality and feasibility of ctDNA for identifying mutations and guiding treatments in NSCLC. Here, we have reviewed recently developed ctDNA detection methods and the importance of sequence analyses of ctDNA in NSCLC.

## Introduction

Lung cancer is the second most common cancer worldwide, responsible for the maximum number of cancer deaths (1). Non-small cell lung cancer (NSCLC) represents approximately 85% of diagnosed lung cancers; lung adenocarcinoma (LUAD) and lung squamous cell carcinoma (LUSC) are the two most common subtypes (2). However, the development and application of precision therapy, including targeted therapy and Immune checkpoint inhibitor (ICI) therapy, have fundamentally altered the management of NSCLC patients. Targeted therapy has shown potential in the treatment of patients with driver gene alterations such as epidermal growth factor receptor (EGFR) mutations, anaplastic lymphoma kinase (ALK) fusions, human epidermal growth factor receptor 2 (HER2) mutations, ROS1 fusions, MET amplification, BRAF mutations, and RET fusions. It is now widely used in daily clinical practice (3). ICI therapy, which suppresses programmed cell death-1 (PD-1) or programmed cell death ligand-1 (PD-L1), has also been successful in prolonging the life of patients (4).

Clinical diagnosis requires a solid biopsy in order to determine tumor histology and staging. Compared with tissue biopsy, liquid biopsy is a non-invasive way to identify patients who might response to therapy, to dynamically monitor treatment effect and to unveil resistance mechanism. Liquid biopsy could typically detect circulating tumor cells (CTCs), circulating tumor DNA (ctDNA), exosomes, microRNAs (miRNA), peripheral blood circulating RNA, tumor-educated blood platelets (TEPs), and circulating tumor vascular endothelial cells (CTECs). ctDNA is one of the most commonly detected biomarkers (5).

Circulating cell-free DNAs (cfDNA) are DNA fragments ranging from 150 to 200 base pairs in length mainly derived from apoptotic or necrotic cells (6). Tumor cells also release circulating tumor DNA (ctDNA), accounting for <0.01% of total cfDNA, which need detection techniques with high sensitivity. Besides the traditional quantitative or real-time PCR (qPCR) and next-generation sequencing (NGS), other recently introduced methods to analyze ctDNA are advanced PCR-based techniques such as digital PCR (dPCR), droplet digital PCR (ddPCR), beads emulsion amplification magnetics (BEAMing), NGS-based techniques such as tagged amplicon deep sequencing (TAM-Seq), safe-sequencing (Safe-Seq), cancer personalized profiling by deep sequencing (CAPP-Seq), and Phased variant enrichment and detection sequencing (PhasED-seq). These will be briefly explained below.

**Figure 1:**
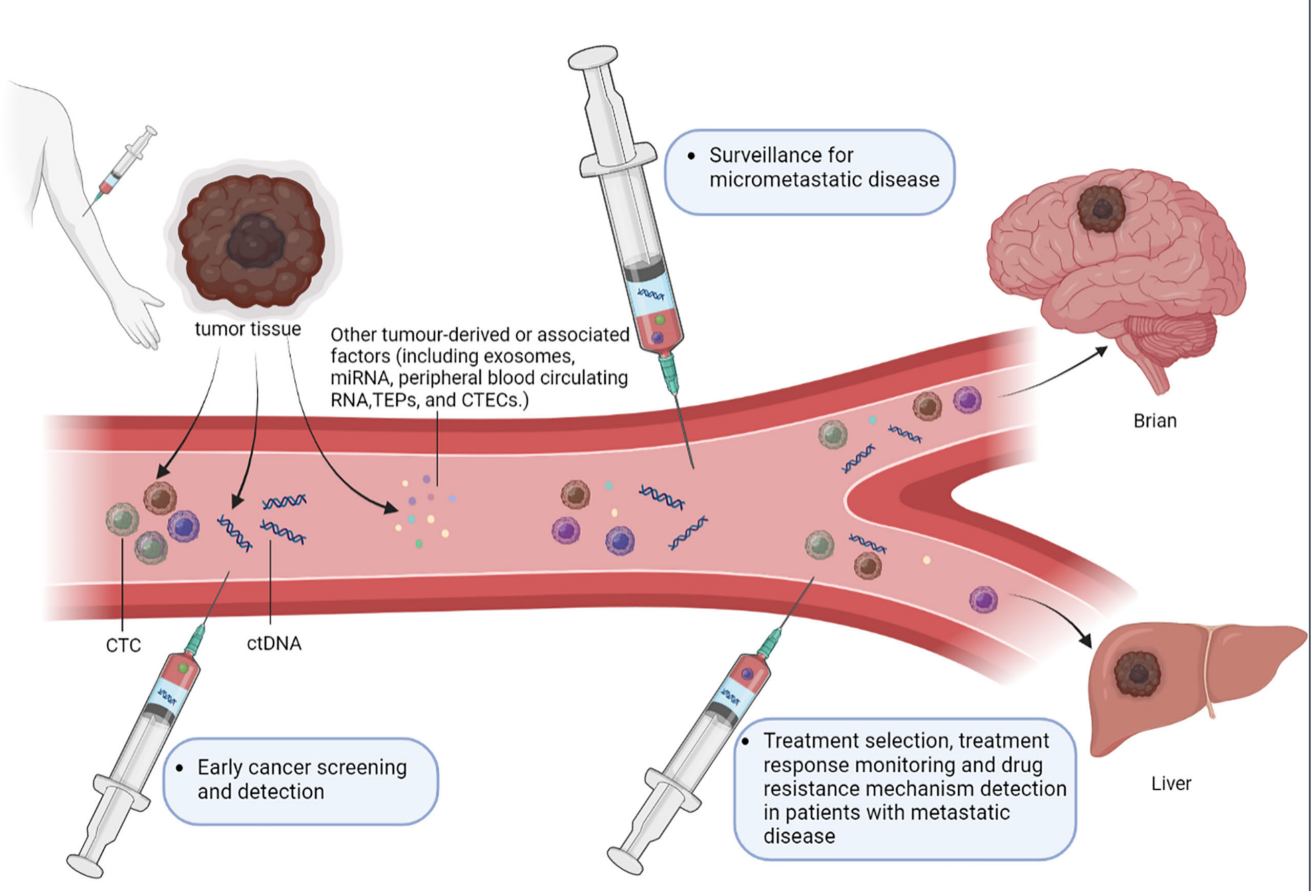
The role of liquid biopsy in MRD detection. Liquid biopsy typically detects circulating tumor cells (CTCs), circulating tumor DNA (ctDNA), exosomes, microRNAs (miRNA), peripheral blood circulating RNA, tumor-educated blood platelets (TEPs) and circulating tumor vascular endothelial cells (CTECs). Liquid biopsy assays of these tumor-derived factors can serve several purposes in the MRD detection. (1) Early cancer screening and detection, liquid biopsy approaches could also be used to further investigate abnormalities detected on imaging examinations. (2) Surveillance for micrometastatic disease following curative-intent treatment of a primary tumor, in order to evaluate the risk of disease recurrence and enable timely management of recurrent disease. (3) Guiding the selection of the most appropriate treatment, monitoring treatment responses and detecting the resistance mechanisms in patients with overt metastatic disease through dynamic characterization of changes in tumor burden and disease biology.

Minimal residual disease (MRD) is a disease status in patients that escapes clinical observation by radiology. In oncology, MRD represents early tumor development and tumor relapse which needs to be urgently detected and assessed (7). In MRD detection, liquid biopsy of these tumor - derived factors plays an important role in clinical application. First of all, liquid biopsy can be used for early cancer screening which lacks detectable abnormalities found by radiology approaches. Secondly, liquid biopsy could monitor micrometastatic disease to assess the risk of disease recurrence after a radical treatment. Finally, the dynamic characterization of tumor burden and disease biological changes could clarify drug resistance mechanisms and guide the treatment strategies. (**Figure 1**) (8). More recently, ctDNA from a liquid biopsy has shown showing their potential to be a reliable plasma-based biomarker for MRD. Quantitative characterization of ctDNA *via* liquid biopsy has been associated with clinical and pathologic features of cancer, including stage, tumor burden, vascularization, and response to therapy. ctDNA can help detect the mutations and activity of different tumor sub-clones,which tissue biopsy cannot because of tumor heterogeneity (9). Moreover, the short half-life of ctDNA ensures that the detection results are in real-time. The molecular precision of longitudinal tumor surveillance *via* serial ctDNA measurement enables the identification of mutations that drive cancer progression and treatment resistance.

In this review, we will introduce several commonly used ctDNA detection approaches and discuss the clinical application of ctDNA-based MRD evalution.

### Recently developed detection techniques

#### PCR assay

Detection and quantitation of specific nucleic acid sequences using PCR is fundamental to a large body of research and a growing number of molecular diagnostic tests. The first generation of PCR users performed end-point analysis by gel electrophoresis to obtain qualitative results. The advent of real-time PCR spawned a second generation. rtPCR is an analogue measurement based on monitoring amplification after each cycle of PCR using fluorescence probes. The point at which the reaction fluorescence crosses an intensity threshold is called the cycle threshold(Ct). As many factors can influence PCR efficiency and hence the Ct value, the accuracy and precision of real-time PCR can vary widely (10). Vogelstein and Kinzler introduced a new form of PCR called digital PCR (dPCR) in 1999 (11). Compared with those conventional PCRs, dPCR partitions samples into multiple parallel quantitative PCR reactions within separate compartments and therefore improves sensitivity, absolute quantification, and rare allele detection (12, 13). However, the large reaction volume and the limited number of compartments to minimize the dimensions of the chip have greatly limited its possible clinical applications (10, 14), and it is believed that ddPCR might overcome these limitations.

Droplet digital PCR (ddPCR) uses aqueous droplets with volumes ranging from a few femtoliters to nanoliters dispersed in oil to compartmentalize PCR reactions, having a theoretically unlimited number of compartments (10). In addition, ddPCR needs only a single reaction tube (10). Nanoliter-sized droplet technology paired with digital PCR (ddPCR) holds promise for highly precise, absolute nucleic acid quantification. Hindson et al. compared the microRNA quantification by ddPCR and real-time PCR which revealed greater precision (coefficients of variation decreased 37–86%) and improved day-to-day reproducibility (by a factor of seven) of ddPCR but with comparable sensitivity (15). Frank Diehl et al. found another droplet-based digital PCR named BEAMing (beads, emulsion, amplification, and magnetics) in 2006. BEAMing couples oligonucleotide primers to beads and distributes beads to compartments. After amplification, every compartment contains a bead binding to thousands of copies of the initial DNA molecule. The DNA can then be released from the beads and analyzed with flow cytometry or optical scanning instruments to locate mutation (16, 17). A recent LungBEAM study demonstrated the great potential of BEAMing in optimizing treatment in patients with advanced NSCLC (18).

Presently, ddPCR and BEAMing are the two most commonly used PCR techniques in clinics to detect ctDNA, their reports must match. Ben O’Leary and his colleagues collected plasma from patients with advanced breast cancer and assessed ESR1 and PIK3CA mutations in ctDNA using both PCR techniques simultaneously. ESR1 mutation was calculated as 24.2% (88/363) with BEAMing and 25.3% (92/363) with ddPCR, (κ = 0.91; 95% CI, 0.85-0.95). The result for PIK3CA mutation was 26.2% (95/363) with BEAMing and 22.9% (83/363) with ddPCR, (κ = 0.87; 95% CI, 0.81– 0.93), showing consistency of results from BEAMing and ddPCR (19). Despite droplet-based digital PCR being highly sensitive, it can only detect known mutations and needs customed assays (20), which restrict its clinical applicability.

#### Targeted next generation sequencing approaches

The development of NGS including whole exome sequencing (WES) and whole genome sequencing (WGS), has facilitated cancer diagnosis over the past decade through samples from a tissue biopsy (21). However, the sensitivity of NGS for single nucleotide variants (SNV) detection is about 4% to 10% (22), which is enough for tissue samples but makes it hard to detect rare mutations in ctDNA for its extremely low percentage in cfDNA. To maintain NGS’s covering broad areas across the genome and meanwhile improve the sensitivity, targeted NGS approaches that detect specific areas of the genome were developed (23).

Forshew et al. established a technique named tagged-amplicon deep sequencing (TAm-Seq; 2012) and successfully applied it to detect ctDNA mutations in patients with metastatic breast cancers and ovary cancers (24). TAm-Seq can detect cancer mutations with allele frequencies as low as 2% and sensitivity and specificity as high as 97% (24). Kinde et al. pioneered Safe Sequencing System (Safe-SeqS; 2011), in which they tagged each template molecule with a 12- or 14- base unique identifier (UID), then amplified the tagged molecules with two cycles of amplicon-specific PCR to create UID families, and sequenced the amplified product redundantly with NGS (25). This redundant sequencing approach makes Safe-SeqS detect rare mutations with high specificity. Moreover, its unique algorithm increases the accuracy of the base calling and reduces the error rate to an average of 2×10^-4^ errors/bp although efficacy is still limited by artifactual mutations occurring during the PCR as well as any residual base-calling errors (25).

Newman et al. developed cancer personalized profiling by deep sequencing (CAPP-Seq; 2014) with ultrasensitive detection of ctDNA. CAPP-Seq utilizes DNA probes to hybridize and capture ctDNAs for its quantification and sequencing. These probes are designed for regions with high driver mutation frequencies in certain cancer types (26). This method can even detect 0.02% of cfDNA and ctDNA in patients with early or advanced stages of NSCLC (26). To further improve the efficiency of ctDNA detection, Newman et al. upgraded CAPP-Seq to integrate digital error suppression-enhanced CAPP-seq (iDES-enhanced CAPP-seq), which tags each template molecule with a UID just like the Safe-seqS to reach a detection limit of 0.001% and a specificity of 96% (27).

Phased variant enrichment and detection sequencing (PhasED-seq; 2021) is the most recent method that uses multiple somatic mutations in individual DNA fragments to improve the sensitivity of ctDNA detection. PhasED-seq can detect less than 0.0001% of tumor DNA, which is better than any earlier approaches (28). David et al. demonstrate that PhasED-seq can meaningfully improve detection of ctDNA in clinical samples both during therapy and before disease relapse. They analyzed serial samples from a participant with stage III lung adenocarcinoma treated with chemoradiotherapy. CAPP-seq and PhasED-seq detected similar ctDNA levels before therapy; however, three samples after treatment initiation had undetectable ctDNA by CAPP-seq before ctDNA re-emerged at the time of biopsy-confirmed recurrent disease. Using PhasED-seq, they observed molecular residual disease in 3/3 (100%) of samples that were undetected by single-nucleotide variants (SNVs), with tumor fraction as low as 0.00016% (28).

Clinical laboratories are increasingly developing and deploying NGS tests, ranging from targeted ‘hotspot’ panels to comprehensive genome-scale platforms. Ahmet et al.developed and implemented MSK-IMPACT, a hybridization capture–based NGS panel with distinct advantages over small panels for detecting all protein-coding mutations, copy number alterations (CNAs), and selected promoter mutations (29). Ivo et al. demonstrate that panel size is a critical parameter that influences confidence intervals (CIs) and cutoff values as well as important test parameters including sensitivity, specificity, and positive predictive value. Panels between 1.5 and 3 Mbp are ideally suited to estimate TMB with small CIs, whereas smaller panels tend to deliver imprecise TMB estimates for low to moderate TMB (0–30muts/Mbp) (30).

### ctDNA and diagnosis of NSCLC

#### Early-stage diagnosis

According to the International Association for the Study of Lung Cancer (IASLC) lung cancer staging project, the 5-year survival of NSCLC diminishes rapidly as the disease stage progresses (82% for stage IA, 52% for stage IIA, 36% for stage IIIA and 6% for stage IV). Thus, the detection of early-stage NSCLC is urgent (31). However, early-stage NSCLC has few radiographic characteristics to be distinguished from benign solitary nodules, so the chances of false positives from radiology approaches are too high. Wong et al. assigned 10,061 candidates to the CANTOS (Canakinumab anti-inflammatory thrombosis outcome study), 71 of them developed lung cancer and each participant had deposited two plasma samples at two different time points during the study; one was at the baseline time point (collected at the beginning of the trial) and the other after the clinical diagnosis of lung cancer. The test of these samples indicated that patients with COSMIC (catalog of somatic mutations in Cancer) ctDNA mutations at baseline exhibited a shorter time to their lung cancer clinical diagnosis (407 days versus 837 days, P=0.011), indicating that mutations in ctDNA might predict an early-stage NSCLC to some extent (32).

A study analyzed ctDNA at different stages of NSCLC utilizing CAPP-seq and found that the diagnostic sensitivity of ctDNA was 64%, 82%, and 100% for tumor stages I, II, and III, respectively. A similar finding was reported from another study (57.9%, 66.7%, and 90% for tumor stages I, II and III, respectively), which implied a correlation between ctDNA levels and tumor volume and outlined the difficulty of early NSCLC detection (33, 34). However, Liang et al. established a method of DNA methylation profiling by high throughput DNA bisulfite sequencing that can distinguish malignant tumors from benign solitary nodules with a sensitivity of 79.5% (63.5%-90.7%) and a specificity of 85.2% (66.3% -95.8%) (35).

### Detection of known mutations

The efficiency of targeted therapy depends on the precise detection of the driver gene mutations. Mack et al. tested plasma samples from 8388 patients and made a plasma-based comprehensive genomic profiling. Driver gene mutations were identified in 48% of patients, including EGFR mutations (26.4%), MET mutations (6.1%), BRAF mutations (2.8%), and fusions (ALK, RET, and ROS1; 2.3%) (36).

Although the golden standard guiding target therapy remains gene mutations detected from a tissue biopsy, non-invasive liquid biopsy utilizing ctDNA is sometimes preferred, and ctDNA increases the chances of identifying several targetable mutations, especially EGFR mutation (37, 38). However, it is crucial to clarify whether the mutations detected from ctDNA agree with those from tumor cell lesions (**Table 1**). A clinical trial study (NCT01203917) aimed to assess the efficacy and tolerability of gefitinib as first-line therapy for common EGFR mutations (19del, L858R, T790M) positive patients in stage III/IV NSCLC. Researchers found EGFR mutations to be similar in tumor and plasma samples (ctDNA) with a sensitivity of 65.7% (95% CI: 55.8–74.7) and a specificity of 99.8% (95% CI: 99.0–100.0) (40), implying that plasma samples are useful to identify patients who might benefit from gefitinib when tumor tissue is unavailable. A similar comparison was designed in the FASTACT-2 study, and the sensitivity and specificity of the mutation detection were 75% and 96%, respectively (43). Cobas EGFR Mutation Test v2, a real-time PCR assay that can identify 42 different EGFR gene mutations, was the first approach approved by FDA to detect EGFR mutations in 2016 (52).

**Table 1:** The mutation results obtained and compared between tumor and plasma DNA samples.

Approach	Gene/TMB	sensitivity	specificity	concordance	reference
ARMS (39)	EGFR (only exon 19 del, L858R mutation and T790M mutation)	65.7% (69/105)	99.8% (546/547)	94.3% (615/652)	(40)
ARMS SURVEYOR (41)	EGFR (only exon 19 del, L858R mutation and T790M mutation)	70% (21/30)	84.6% (11/13)	74% (32/43)	(42)
Cobas	41 EGFR mutations	75% (72/96)	96.5% (137/142)	87.8% (209/238)	(43)
real-time PCR	exon 19 del	64% (112/175)	96.4% (149/153)	79.6% (261/328)	(44)
	L858R mutation	55.1% (70/127)	99.5% (200/201)	82.3% (270/328)	
	T790M mutation	0 (0/1)	99.7% (326/327)	99.4% (326/328)	
ddPCR	EGFR (only exon 19 del, L858R mutation and T790M mutation)	70% (182/260)	93.9% (123/131)	78.0% (305/391)	(45)
BEAMing	EGFR (only exon 19 del and L858R mutation)	70.9% (78/110)	/	/	(18)
PNA-based real-time PCR	47 EGFR mutations	66.7% (34/51)	87.4% (125/142)	82.0% (159/193)	(46)
BEAMing	EGFR (only exon 19 del and L858R mutation)	81.5% (44/54)	/	/	(47)
	EGFR T790M mutation	83.7% (41/49)	/	/	
PNA-based real-time PCR	EGFR (only exon 19 del, L858R, G719X and L861Q mutation)	59.3% (48/81)	92.0% (230/250)	84.0% (278/331)	(48)
NGS	ALK fusions	54.2% (13/24)	99.8% (472/473)	97.6% (485/497)	(49)
capture-based NGS	ALK fusions	79.2% (19/24)	100% (36/36)	91.7% (55/60)	(50)
^a^Targeted NGS	bTMB	50.7% (77/152)	86.0% (172/200)	70.7% (249/352)	(51)

a The cut-off of bTMB is 20 mut/Mb and that of tTMB is 10 mut/Mb.

Pertaining to the oncogenic fusions, ctDNA reflects a high similarity with those detected in tissue samples. Horn et al. analyzed ALK fusions in tumor and plasma samples and found a concordance of 91% (20/22) between them (53). Wang et al. also detected ALK fusions in ctDNA from 19 out of 24 patients with ALK fusions in their tumor tissue, demonstrating a sensitivity of 79.2% (95%CI: 57.9%-92.9%). They could not detect ALK fusions in ctDNA from 36 patients without ALK fusions in their tumor tissue, implying that the specificity of the method was 100% (50). Plasma ROS1 fusions analysis also showed a 100% concordance with those observed in the tissue samples (54).

Vansteenkiste et al. found that the similarity in PIK3CA mutations between tissue and ctDNA samples was 55.3%. However, the concordance was 81.8% (9 of 11 samples) between ctDNA and metastatic tissue samples, compared with 44.4% (12 of 27 samples) between ctDNA and primary tissue, implying that ctDNA PI3K pathway mutations were more correlated with metastatic lesions than with primary tumor (55).

However, despite the high sensitivity of methods for detecting mutations in ctDNA, it is wise to retest tissue samples if the result is negative (56).

It is noteworthy that MRD detection can also be confounded by clonal hematopoiesis of indeterminate potential (CHIP). CHIP arises when age-dependent mutations accumulate in hematopoietic progenitor cells, leading to the formation of a genetically distinct subpopulation that contributes disproportionately to the population of mature blood cells. These distinct subclones have driver mutations and have been implicated in hematologic diseases. In the measurement of ctDNA, CHIP can result in false-positive results due to detection of non-reference variants in the blood plasma, which is especially problematic when the ctDNA mutant allele fraction is low in the setting of MRD detection. Thus, CHIP must be properly accounted for in order to specifically measure ctDNA, such as by sequencing matched PBMCs to similar depth, especially when using ultra-sensitive assays that are capable of achieving detection of low mutant allele fraction variants (57).

### ctDNA and Treatment of NSCLC

#### Relapse after operation

The relapse after curative-intent resection has confused surgeons for years and ctDNA might be an early predictor of it. The DYNAMIC prospective study tested plasma ctDNA collected from 36 patients that underwent curative-intent lung resections 7 times, immediately before surgery (time A), after tumor resection [time B (5 minutes), time C (30 minutes), and time D (2 hours)] and after surgery [time P1 (1 day), time P2 (3 days), and time P3 (1 month)]. A rapid decrease in the content of ctDNA was found after the curative-intent resection (the mean mutant allele fraction at times A, B, C, and D was 2.72%, 2.11%, 1.14%, and 0.17%, respectively) which implied that the half-time of ctDNA is short and there is an association between ctDNA and tumor volume. In addition, the detection of ctDNA at time P2 (278 days versus 637 days, P=0.002) and time P3 (295 days versus 662 days, P=0.003) rather than time P1 (528 days versus 543 days, P=0.657) was negatively correlated with recurrence free survival (RFS) of patients; similar correlations were observed between ctDNA detection and overall survival (OS) (58).

Xia et al. analyzed ctDNA in another prospective, multicenter study (LUNGCA-1; 2021) on NSCLC surgery patients. They found that detectable ctDNA before operation (RFS; HR=4.2, 95%CI: 2.6-6.7; P < 0.001) or at 3 days and/or 1 month after operation (RFS; HR=11.1, 95%CI: 6.5-19.0; P < 0.001) was a robust predictor for relapse in patients with stage I–III NSCLC. Moreover, ctDNA status was tightly associated with the benefit of postoperative adjuvant therapy — ctDNA-positive patients who received adjuvant therapies had improved RFS over those that did not receive (RFS; HR=0.3, 95%CI: 0.1-0.8; P=0.008), while ctDNA-negative patients receiving adjuvant therapies had impaired RFS than those that did not receive (RFS; HR=3.1, 95%CI: 1.7-5.5; P < 0.001) (7).

Chaudhuri et al. prespecified “MRD landmark” as the ctDNA status following the first phlebotomy of curative-intent resection and within 4 months from the end of therapy, progression at 36 months after the MRD landmark was 100% and 7% in patients with detectable and undetectable ctDNA MRD (HR=43.4, 95%CI=5.7–341; P < 0.001), respectively (59).

Yilong Wu et al. elucidated the role of MRD monitoring in patients with stage I to IIIA NSCLC after definite surgical resection. Patients with undetectable MRD at landmark or longitudinal time points had better disease-free survival (DFS) than those with detectable MRD [landmark: unreached vs. 12.1 months (4.7–19.5); HR = 0.08; 95% CI, 0.02–0.33; longitudinal: unreached vs. 15.9 months (13.8–18.0); HR = 0.02; 95% CI, 0.01–0.05]. 96.8% of patients with longitudinal undetectable MRD were still disease-free at the last follow-up and had nothing to do with the clinical stage, thus it may represent the potentially cured population, which has important application value for the treatment of early lung cancer in the future. Because MRD status reflected the tumor load, adjuvant therapy was found to confer a survival benefit for patients with detectable MRD (P = 0.022; HR = 0.34; 95% CI, 0.12-0.88) (60).

#### Development of resistance due to targeted therapy

Driver gene mutations might exhibit changes during tumor development or treatment that can lead to resistance to the drugs, which limits the long-term use of targeted therapy. Thus, the new driver gene mutations need to be detected through a re-biopsy. Liquid biopsy can specifically detect the new gene mutations, and this can be used to predict targeted therapy resistance development in patients.

Approximately half or more NSCLC patients with EGFR mutations who develop resistance to the first- and second-generation EGFR-tyrosine kinase inhibitors (TKI) will develop a secondary EGFR T790M mutation in the tumor (61). Additionally, the ctDNA T790M mutation is more likely to be seen in patients with an initial EGFR del19 mutation compared with the EGFR L858R mutation (62). ctDNA analysis may help in predicting such resistance and directing the use of subsequent therapy such as the use of osimertinib, an oral, irreversible third-generation EGFR-TKI, approved by FDA in 2015 (42, 63). LiquidLung-O-Cohort 2 study screened ctDNA from patients with EGFR T790M mutation with a detection sensitivity of 56.8% (64). Serial monitoring of EGFR mutation in ctDNA is able to detect EGFR T790M mutation much earlier (range: 15-344 days) than clinical manifestation of the disease progression (65). However, since osimertinib is considered standard first-line therapy for NSCLC patients with EGFR mutations (66), some patients on osimertinib would inevitably develop new mutations (detected in ctDNA), including EGFR C797S mutation, MET amplification, HER2 exon 20 insertions, BRAF^V600E^ mutation, PIK3CA mutation, and EGFR amplification and thus be resistant to the drug (67–71).

Dagogo et al. analyzed plasma and tumor samples from patients with progressed ALK-positive NSCLC treated with alectinib (2019). There was no difference in ALK mutation frequency (67% versus 63%), but ctDNA was more likely to harbor ≥2 ALK mutations (24% versus 2%, P=0.004). However, ALK L1196M, a gatekeeper mutation that leads to resistance to crizotinib, showed little prevalence between tumor DNA and ctDNA (2% versus 22%, P=0.008), which implies that ctDNA can predict ALK-TKI resistance sometimes. A similar phenomenon was found in those with lorlatinib, showing a promoted acquisition of ALK resistance mutations after sequential treatment with increasingly potent ALK-TKIs (72). Secondary ALK mutations such as ALK G1202R, ALK G1269A, and ALK L1196M were found in ctDNA through NGS, causing genetic resistance to first- and second-generation ALK-TKIs (50, 72–74).

#### Prognosis and treatment response after target therapy

ctDNA is commonly used to monitor the benefit of the treatment and predict progression *via* liquid biopsy. Several studies have found a significant association between the quantitative changes in ctDNA, the response of cancer to the targeted therapy, and the prognosis of NSCLC (**Table 2**).

**Table 2:** ctDNA as a treatment response predictor.

Patients	Treatment	Standard	Test time	Approach	Findings	reference
Stage I– IIIA	curative-intent resections	RFS	Day 3	NGS	278d vs. 637d for ctDNA detected and EGFR undetected patients (HR=7.55; 95% CI: 2.09–27.27, P=0.002)	(58)
		OS			434d vs. 720d for ctDNA detected and EGFR undetected patients (HR=14.22; 95% CI: 1.58–128.15, P=0.018)	
ctDNA EGFR mut^+^ at baseline	gefitinib	PFS	Week 8	ddPCR	11.0m vs. 2.1m for ctDNA EGFR mut^-^ and EGFR mut^+^ patients (HR=0.14; 95% CI: 0.08–0.23, P < 0.0001)	(45)
ctDNA EGFR mut^+/-^ at baseline	erlotinib	PFS	Week 8 (Cycle 2)	Cobas	11.0m vs. 5.7m for ctDNA EGFR mut^-^ and EGFR mut^+^ patients (HR=0.28; 95% CI: 0.15–0.52, P < 0.0001)	(75)
		OS			30.1m vs. 15.8m for ctDNA EGFR mut^-^ and EGFR mut^+^ patients (HR=0.35; 95% CI: 0.19–0.64, P < 0.0001)	
ctDNA EGFR mut^+^ at baseline	erlotinib	PFS	Cycle 3	Cobas	12.0m vs. 7.2m for ctDNA EGFR mut^-^ and EGFR mut^+^ patients (HR=0.32; 95% CI: 0.21–0.48, P < 0.0001)	(43)
		OS			31.9m vs. 18.2m for ctDNA EGFR mut^-^ and EGFR mut^+^ patients (HR=0.51; 95% CI: 0.31–0.84, P=0.0066)	
ctDNA T790M mut^+^ at baseline, progress on 1/2G EGFR-TKI	osimertinib	PFS	Cycle 4	ddPCR	4.9m vs. 15.9m for ctDNA EGFR MF-high and EGFR MF-low patients (HR=4.54; 95% CI: 2.23–9.23, P < 0.0001)	(71)
				Cobas	6.3m vs. 17.2m for ctDNA EGFR MF-high and EGFR MF-low patients (HR=2.87; 95% CI: 1.52–5.42, P=0.0012)	
				NGS	4.3m vs. 14.5m for ctDNA EGFR MF-high and EGFR MF-low patients (HR=6.00; 95% CI: 2.87–12.55, P < 0.0001)	
ctDNA EGFR mut^+^ at baseline	osimertinib + bevacizumab	PFS	Week 6	ddPCR	16.2m vs. 9.8m for ctDNA EGFR mut^-^ and EGFR mut^+^ patients P=0.04	(76)
		OS			NR vs. 10.1m for ctDNA EGFR mut^-^ and EGFR mut^+^ patients P=0.002	
StageIIIB/IV, progressed following two or more systemic treatments	durvalumab	PFS	Week 6	targeted NGS	5.6m vs. 1.9m for ctDNA dVAF<0 and dVAF≥0 HR 0.26 (95% CI, 0.12–0.54).	(77)
		OS			NR vs. 8.7m for ctDNA dVAF<0 and dVAF≥0 HR 0.23 (95% CI, 0.09–0.61).	

High plasma cfDNA is associated with poor OS (16.0 months versus 28.6 months, P=0.030) and increased risk of death (HR=1.23, 95% CI: 1.01-1.50; P=0.045) (78). Bordi et al. defined a cut-off of 2200 copies/ml generated by means of ROC analysis and found a lower number of mutations (< 2200 copies/mL; at baseline) are associated with better progression free survival (PFS; 17.8 months versus 4.3 months, p=0.022) and OS (23.6 months versus 7.7 months, p= 0.016) (79).

Baseline EGFR T790M mutation detection in ctDNA might correlate with a larger baseline tumor size (56 mm for T790M (+) versus 39 mm for T790M (-); P < 0.0001) and a higher probability of extra thoracic metastasis [58% M1b for T790M (+) versus 39% M1b for T790M (-); P = 0.002] (80). Moreover, tissue T790M positive patients without detectable T790M mutation in the ctDNA had a longer PFS, which might be attributed to a lower tumor burden (80).

Identification of EGFR mutation in ctDNA before the start of the treatment procedure helps to select patients who might benefit from EGFR-TKI treatment, and monitoring ctDNA consistently for further EGFR mutation helps to predict the outcome of current treatment and the patient’s prognosis (45, 81). In patients treated with erlotinib and assessed to be stable disease (SD), undetectable ctDNA at week 8 is correlated with survival improvement (PFS: HR=0.27, 95%CI: 0.13-0.57, p<0.0001; OS: HR=0.40, 95% CI 0.20–0.80, p=0.009) (75). In NSCLC patients with progression after EGFR-TKI therapy, chest- or brain-limited disease has a significantly higher rate of ctDNA T790M mutation than the others (P<0.001). This showed that both ctDNA T790M mutation status and TKI treatment failure can predict prognosis (82). Furthermore, the persistence of EGFR mutation in ctDNA at 6 weeks in patients treated with osimertinib was associated with shorter PFS (9.8 months versus 16.2 months, P=0.04) (76), while loss of EGFR exon 19 deletion or L858R mutation post-treatment appears to correlate with longer PFS (14.7 months versus 5.5 months) (67). The monitering of ctDNA for EGFR mutations in NSCLC patients at treatment cycle 4 is optimal for predicting the treatment outcomes for patients receiving osimertinib (71).

Patients with EML4-ALK fusion variants 1 detected in ctDNA at baseline exhibited longer PFS than those with EML4-ALK fusion variants 3 [8.2 months (95% CI: 2.1–11.7) versus 1.9 months (95% CI: 1.8-not estimable)] (53). In the ALTA-1L study, researchers found detectable baseline EML4-ALK fusion variant 3 rather than variant 1 in ctDNA, which was associated with poor PFS in patients treated with ALK TKI [crizotinib: HR: 3.42 (1.56–7.50), P=0.002; brigatinib: HR: 2.45 (1.07–5.60), P=0.033] (83).

#### Prognosis and treatment outcome after immunotherapy

Even though long-term positive responses have been observed in NSCLC patients receiving ICI therapy, the majority of them become refractory with an eventual unfavorable clinical outcome (84). Rapid as well as sensitive detection of dynamic changes in the ctDNA might help to identify NSCLC patients and plan appropriate immunotherapy for them (85). (**Table 2**)

Goldberg et al. defined ctDNA response as a >50% decrease in mutant allele fraction from its baseline (2018). In ICI therapy receiving patients with metastatic NSCLC, ctDNA response greatly agreed with the radiographic response (κ=0.753), and benefits could be assessed faster from ctDNA than radiographically (median 24.5 days versus median 72.5 days). Additionally, a ctDNA response is associated with long-term treatment benefit (205.5 days versus 69 days; P<0.001) as well as better prognosis (PFS: HR=0.29, 95%CI: 0.09–0.89, P=0.03; OS: HR=0.17, 95%CI: 0.05–0.62; P= 0.007) (86). Similar to target therapy, patients with undetectable levels of ctDNA were demonstrated to have significantly longer PFS (P=0.001) and OS (P=0.008) compared with those with no evidence of ctDNA clearance (85). Hellmann et al. tested ctDNA of 31 patients with advanced NSCLC and had achieved long-term benefit from ICI therapy (PFS≥12 months) at a median time of 26.7 months after the initiation of therapy. They found 25/27 (93%) patients with ctDNA negative remained progression-free, while in 4 patients with ctDNA positive, the desease eventually progressed (87).

Nabet et al. established an approach named DIREct-On (Durable Immunotherapy Response Estimation by immune profiling and ctDNA- On-treatment) to predict whether patients with NCSLC would show durable clinical response to ICI therapy. DIREct-On incorporated pre-treatment ctDNA and immune profiling with early on-treatment ctDNA response assessment and could get an accuracy of 92% to identify the potential patients obtaining benefit (88).

A high tumor mutation burden (TMB) and microsatellite instability (MSI) are demonstrated to correlate with a better response to immunotherapy in NSCLC (89–91).,TMB is measured from tumor tissue traditionally. Si et al. measured and compared TMB from tissue (tTMB) and plasma (bTMB) samples and found a positive correlation between bTMB (using a cutoff of 20 mut/Mb) and tTMB (using a cutoff of 10 mut/Mb) values (P<0.0001, χ^2^ test). They also found that higher bTMB was also associated with the clinical benefits of immunotherapy (51). When the bTMB cut-off point was set to 6, patients with higher bTMB showed superior PFS (NR versus 2.9 m; HR=0.39, 95%CI: 0.18-0.84, P=0.01) (92). Goldberg et al. found that bTMB is independently predictive of the immunotherapy outcome benefits without association with high PD-L1 expression. They further discovered that bTMB=16 mut/Mb is a clinically meaningful cut-off point in NSCLC and patients with bTMB≥16 mut/Mb benefited from a second-line immunotherapy rather than chemotherapy (PFS was 4.2m in the atezolizumab arm and 2.9m in the docetaxel arm, HR=0.57, 95%CI: 0.33–0.99; OS was 13.0m and 7.4m, HR=0.56, 95%CI: 0.31–0.99) (93). Similarly, Georgiadis et al. defined ctDNA bTMB≥10 mut/Mb of the whole exome as bTMB-high and demonstrated that bTMB-high before immunotherapy predicted a better PFS (HR=0.23, 95% CI, 0.07–0.63, P=0.003) and OS (HR=0.26, 95% CI, 0.08–0.72, P=0.008) in pan-cancer. Additionally, patients with blood MSI also had a better PFS (HR=0.21, 95% CI, 0.08–0.54, P=0.001) and OS (HR=0.41, 95% CI, 0.16–1.05, P=0.063) than those with microsatellite stability (MSS) (94).

Variant allele frequencies (VAF) in ctDNA can also predict immunotherapy response as an alteration of TMB. Patients with decreased VAF at week 6 of the treatment had a mean reduced tumor volume by 39%, while those with a high VAF had a mean increased tumor volume by 36% (77). Additionally, a decrease of VAF at week 6 of the immunotherapy implied a longer PFS and OS (77).

## Discussion and limitation

Several clinical trials have demonstrated definite correlations between ctDNA levels and NSCLC patients’ medical status, including tumor sizes, recurrence post operations, choice of treatments, treatment response and prognosis. Thus, ctDNA could help to guide clinicians in selecting appropriate therapies for each patient: whether to utilize adjuvant therapies after curative-intent resections, how to select a treatment that could benefit a patient maximumly and how to evaluate treatment efficiency and diagnose drug resistance on time. The short half-life of ctDNA enables it to exhibit a real-time status of a dynamic disease and overcome temporal tumor heterogeneity. Additionally, as ctDNA could represent spatial heterogeneity better than primary tumors or metastatic lesions biopsy, ctDNA has an inherent advantage in monitoring a patient’s condition. Undoubtedly, ctDNA can serve as a predictor of MRD and can be frequent applied in the medical management of NSCLC patients. At the same time, there were a lot of research about MRD in predicting the recurrence trajectory of early lung cancer and the curative effect of consolidation immunotherapy, which show great clinical application prospect.

Based on this, we put forward some ideas. For lung cancer patients with driver gene mutations after radical resection, ctDNA-based MRD monitoring to guide the use of targeted drugs, rather than continuous drug use mode, can theoretically delay the development of drug-resistant clones of tumor targeted therapy, thereby delaying drug resistance? At the same time, the treatment burden of patients can be reduced; For patients with inoperable locally advanced NSCLC, after radical therapy, ctDNA-based MRD monitoring can be used to guide immunodrug maintenance therapy, which can not only predict the population benefiting from ICI consolidation therapy, but also reduce the treatment burden. Starting from a population of patients with advanced targeted therapy, MRD monitoring should be used to guide the use of targeted drugs after patients have achieved complete remission or local treatment for oligometrics.

However, some limitations can hamper the wide use of ctDNA. Early-stage NSCLC remains tough to be detected by ctDNA mainly because of its extremely low concentrations due to small tumor sizes. Thus, highly sensitive methods need to be developed. Moreover, most of the trials that utilize ctDNA to plan the targeted therapy and predict treatment response or prognosis focused on the most common driver gene mutation, EGFR mutations. The use of ctDNA in patients with ALK fusions, MET amplification, HER2 mutations, and other rare mutations still needs to be studied in detail.

To conclude, a transformation in the management of NSCLC patients by analyzing ctDNA is afoot.

## Author contributions

XF, SY, YJ, YX, KL drafted the manuscript, conceived and designed the study, and accomplished the revision of manuscript for important intellectual content. KL obtained funding. All authors contributed to the article and approved the submitted version.

## Funding

This study is supported by the National Natural Science Foundation of China (81902327, 82172708); and the Jiangsu National Natural Science Foundation (BK20191064).

## Conflict of interest

The authors declare that the research was conducted in the absence of any commercial or financial relationships that could be construed as a potential conflict of interest.

## Publisher’s note

All claims expressed in this article are solely those of the authors and do not necessarily represent those of their affiliated organizations, or those of the publisher, the editors and the reviewers. Any product that may be evaluated in this article, or claim that may be made by its manufacturer, is not guaranteed or endorsed by the publisher.
